# Dermatologic Manifestations of Noninflammasome-Mediated Autoinflammatory Diseases

**DOI:** 10.1016/j.xjidi.2022.100176

**Published:** 2022-12-16

**Authors:** Dörte Symmank, Carina Borst, Mathias Drach, Wolfgang Weninger

**Affiliations:** 1 Department of Dermatology, Medical University of Vienna, Vienna, Austria

**Keywords:** AID, autoinflammatory disease, ANCA, antineutrophil cytoplasmic antibody, AOSD, adult-onset Still disease, BASDAI, Bath Ankylosing Spondylitis Activity Index, CANDLE, chronic atypical neutrophilic dermatosis with lipodystrophy and elevated temperature, CAPS, cryopyrin-associated periodic syndrome, CRD, cysteine-rich domain, DIRA, deficiency of IL-1RA, DITRA, deficiency of IL-36RA, ER, endoplasmic reticulum, ESR, erythrocyte sedimentation rate, FMF, familial Mediterranean fever, MAS, macrophage activation syndrome, M-CSF, macrophage colony-stimulating factor, NET, neutrophil extracellular trap, NOS, nitrous oxide, NSAID, nonsteroidal anti-inflammatory drug, NUD, neutrophilic urticarial dermatosis, PFAPA, periodic fever, aphthous stomatitis, pharyngitis, and adenitis, PRAAS, proteosome-associated autoinflammatory disease, PKR, protein kinase R, SAPHO, synovitis, acne, pustulosis, hyperostosis, osteitis syndrome, SAVI, STING-associated vasculopathy with onset in infancy, SchS, Schnitzler syndrome, STAT, signal transducer and activator of transcription, sTNFR, soluble TNF receptor, Th17, T helper 17, TNFR, TNF receptor, TRAPS, TNF receptor‒associated autoinflammatory disease, VAS, Visual Analog Scale

## Abstract

Autoinflammatory diseases (AIDs) arise from disturbances that alter interactions of immune cells and tissues. They give rise to prominent (auto)inflammation in the absence of aberrant autoantibodies and/or autoreactive T cells. AIDs that are predominantly caused by changes in the inflammasome pathways, such as the NLRP3- or pyrin-associated inflammasome, have gained substantial attention over the last years. However, AIDs resulting primarily from other changes in the defense system of the innate immune system are less well-studied. These noninflammasome-mediated AIDs relate to, for example, disturbance in the TNF or IFN signaling pathways or aberrations in genes affecting the IL-1RA. The spectrum of clinical signs and symptoms of these conditions is vast. Thus, recognizing early cutaneous signs constitutes an important step in differential diagnoses for dermatologists and other physicians. This review provides an overview of the pathogenesis, clinical presentation, and available treatment options highlighting dermatologic aspects of noninflammasome-mediated AIDs.

## Introduction

Most patients with autoinflammatory diseases (AIDs) present with recurrent flares of fever, showing several additional systemic and cutaneous signs and symptoms. Because skin findings encompass a wide range of possible rashes and often overlap with nonautoinflammatory conditions, physicians encountering AIDs should be aware of common skin lesions in the disease course. In addition, knowledge of the underlying pathogenic mechanism and clinical presentation shortens the time of diagnosis and therefore lessens the disease burden for patients. In this paper, we present a comprehensive review of cutaneous lesions seen in noninflammasome-mediated AIDs and discuss the general clinical presentation of affected patients as well as the underlying mechanism leading to the pathophysiology of the disease.

## Cutaneous Signs seen in Noninflammasome-Mediated AIDs

**Figure 1:**
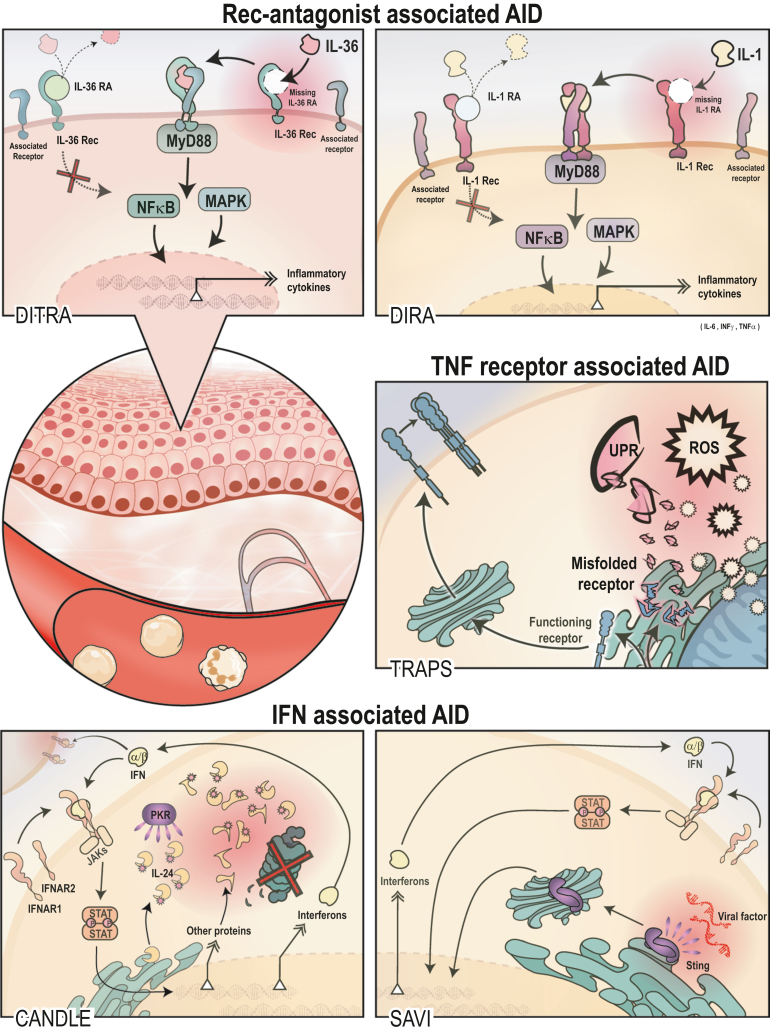
**Cytokine (receptor) associated AIDs.** The circle in the middle shows the skin (epidermis + dermis) as well as immune cells traveling in the vessels. Top panel: IL-1RA family‒associated disease includes DITRA (left top panel), mainly affecting keratinocytes, and DIRA (right top panel), affecting myeloid cells as well as other cell types. In DITRA, IL-36RA hinders the binding of IL-36 to the receptor, which provides a mechanism regulating proinflammatory cytokine release. Without IL-36RA, IL-36 binds to the receptor and signals in a MyD88-dependent manner. Proinflammatory cytokine production is elevated through the NF-kB and MAPK pathways. A similar mechanism is seen for the loss of IL-1RA in DITRA. Middle panel: TNF receptor‒associated disease TRAPS (right middle panel) shows the upregulation of ROS through processes inside the mitochondrion as well as the UPR induced by the accumulation of misfolded (red shining receptors with teal filling) TNF receptors. Other possible theories related to the pathophysiology of TRAPS are explained in detail in Figure 2. Bottom panel: Interferonopathies show CANDLE (left bottom panel) and SAVI (right bottom panel). CANDLE shows a failed proteasomal degradation of ubiquitinated proteins, which is sensed by protein kinase R owing to the accumulation of IL-24. Inflammation leads to the damage of proteins and further to an upregulated de novo synthesis of proteins and type 1 IFN (mainly IFN-α/γ), which leads to a vicious cycle of autostimulation. It also stimulates surrounding cells. SAVI shows a constitutional activation of the viral sensor STING, which is bound to the endoplasmic reticulum. Activated STING wanders through the Golgi apparatus near the nucleus, upregulating type 1 IFNs production through IRF3. In both cases, type I IFN binds to the IFN receptor and signals through the Jak‒STAT pathway to amplify the inflammatory response. AID, autoinflammatory disease; CANDLE, chronic atypical neutrophilic dermatosis with lipodystrophy and elevated temperature; DIRA, deficiency of IL-1RA; DITRA, deficiency of IL-36RA; SAVI, STING-associated vasculopathy with onset in infancy; STAT, signal transducer and activator of transcription; TRAPS, TNF receptor‒associated autoinflammatory disease; UPR, unfolded protein response.

AIDs predominantly driven by gain-of-function mutations in one of the inflammasome platforms (e.g., NLRP3 inflammasome leading to cryopyrin-associated periodic syndrome [CAPS] or the pyrin inflammasome leading to familial Mediterranean fever [FMF]) commonly show high levels of IL-1β resulting from the immediate impact of the mutation on the function of the inflammasome (Sangiorgi and Rigante, 2022). We recently reviewed cutaneous signs seen in inflammasome-mediated AIDs (Borst et al., 2022). Although the inflammasome can be triggered through various factors, noninflammasome-mediated AIDs show no direct mutation in the building blocks of the inflammasome. Symptoms seen in noninflammasome-mediated AIDs are instead underlined by the disruption of various cytokine signaling pathways or receptors with effects on tissue-resident cells as well as infiltrating immune cells (Figure 1). The commonly observed overproduction of proinflammatory cytokines may have detrimental effects on tissues. The TNF, IFNs, and the IL-1 family are three of the key cytokine pathways involved in the pathophysiology of AIDs. Because AIDs present with diverse symptoms, diagnosis requires an interdisciplinary workup of the patient. Skin findings can range from migratory, erythematous patches and plaques (TNF receptor‒associated autoinflammatory disease [TRAPS]) over a unique combination of progressive lipodystrophy, violaceous plaques with raised borders, and violaceous swellings of the lips and eyelids (chronic atypical neutrophilic dermatosis with lipodystrophy and elevated temperature [CANDLE] and generalized erythema with studded pustules—deficiency of IL-36RA [DITRA]) to urticaria-like lesions (Schnitzler syndrome [SchS]) (Table 1). Histopathological findings of skin biopsies can be found in Table 2. We aim to highlight the dermatologic aspects of these diseases and provide a working tool for physicians in clinical skin examination.

**Table 1:** Cutaneous Signs in Noninflammasome-Mediated AIDs

Cytokine (Receptor) Associated AIDs	Cutaneous Sign	Localization	Onset	Duration of Flare	Trigger of Flare	Class^1^
TRAPS	Migratory erythematous plaques and patches; periorbital edema	Trunk, extremities, face	Childhood	2‒3 weeks	Unprovoked or by stress, exercise, infection, injury, hormonal changes	(1)
IFN associated						
CANDLE	Erythematous to violaceus, edematous maculae; erythematous or violaceous, pruritic plaques; edema; lipodystrophy	Acra; face, trunk, extremities; lips, eyelids; initially in the face, then progression to trunk and extremities	Neonatal period	1 day	Stress, cold temperature, infections	(1)
SAVI	Erythematous to violaceous, infiltrated plaques, ulcers, tissue loss; erythema, ear, and saddle-nose deformities	Acra, extremities; face	Infancy	Undefined	Cold temperature, unprovoked	(5)
IL receptor signaling defects						
DIRA	Erythematous plaques with pustules, nail dystrophy	Generalized, nails	Birth, neonatal period	Undefined	Trauma (pathergy), unprovoked	(3)
DITRA	Erythematous plaques with pustules; nail dystrophy; benign migratory glossitis, scrotal tongue	Generalized; nails, tongue	Neonatal period, infancy, childhood, adulthood	Few days to 1 week	Infection, stress, drug intake, drug withdrawal, menstruation, pregnancy, red wine, surgery	(3)

**Other or Polygenic Autoinflammatory Diseases**	**Cutaneous Sign**	**Localization**	**Onset**	**Duration of Flare**	**Trigger of Flare**	**Class**^1^
SAPHO	Pustules; acne	Palms, soles; face, neck, trunk, and extremities	Adulthood	Undefined	Unknown	(3)
AOSD	Evanescent, salmon-colored macular or maculopapular rash; papules, plaques, (erythematous, urticaria-like, heliotrope-like, lichenoid)	Trunk, extremities; head, trunk, extremities	Adulthood	Few hours	Infection, unprovoked	(1)
SchS	Urticarial rash	Trunk, extremities, rarely head or neck	Adulthood	12-48 hours	Stress, physical work, alcohol, spicy food, cold or hot temperatures	(2)
PFAPA	Aphtae	Enoral	Infancy, childhood	1‒10 days	unknown	(9)

Abbreviations: AID, autoinflammatory syndrome; AOSD, adult-onset Still disease; CANDLE, chronic atypical neutrophilic dermatosis with lipodystrophy and elevated temperature; DIRA, deficiency of IL-1RA; DITRA, deficiency of IL-36RA; PFAPA, periodic fever, aphthous stomatitis, pharyngitis, and adenitis; SAPHO, synovitis, acne, pustulosis, hyperostosis, osteitis syndrome; SAVI, STING-associated vasculopathy with onset in infancy; SchS, Schnitzler syndrome; TRAPS, TNF receptor‒associated autoinflammatory disease.

The table provides an overview of discussed AIDs. The onset and localization of cutaneous lesions as well as the duration and the trigger of flare-up of the disease are listed. Each AID is sorted into the classification schemata proposed by Figueras-Nart et al. (2019).

1 Class: classification based on Figueras-Nart et al. (2019): (1) maculopapular rashes or inflammatory plaques; (2) urticarial rashes; (3) pustular, pyogenic, or neutrophilic dermatosis-like rashes; (4) panniculitis or subcutaneous nodules; (5) vasculitis or vasculopathy; (6) hyperkeratotic lesions; (7) hyperpigmented lesions; (8) bullous lesions; and (9) aphthous lesions.

**Table 2:** Histopathological Presentation of Noninflammasome-Mediated Autoinflammatory Diseases

Autoinflammatory Disease	Histopathological Signs
TNF associated	
TRAPS	No specific pattern can be observed histopathologically; findings are rather unspecific. Most frequently, a perivascular infiltrate affecting the upper and the mid-dermal plexus can be observed, composed of lymphocytes and histiocytes (Schmaltz et al., 2010).
IFN associated	
CANDLE	The entire dermis shows a perivascular and interstitial infiltrate consisting of mononuclear cells, neutrophils, eosinophils, and atypical myeloid cells, which could be proven (characterized) by immunohistochemistry (Torrelo et al., 2015).
SAVI	In early lesions, dermal capillaries show signs of vasculitis with no evidence of affection of medium-sized vessels. Older lesions show signs of a vaso-occlusive disease (Liu et al., 2014).
IL-1 family associated	
DIRA	Skin biopsy reveals a subcornear pustule accompanied by a suprabasal acantholysis. In addition, neutrophil eccrine hidradenitis could be observed (Minkis et al., 2012).
DITRA	Irregularly arranged acanthosis, hyperkeratosis, and parakeratosis associated with intraepidermal spongiform pustules. Mixed superficial perivascular infiltrate (Marzano et al., 2018).
Polygenic	
SAPHO	Two main patterns can be recognized: (i) palms and soles show a psoriasiform epidermal hyperplasia with intraepidermal abscess formation accompanied by a superficial perivascular chronic inflammatory infiltrate and (ii) head and neck as well as trunk show a neutrophil-rich folliculitis and perifolliculitis (DermNet, 2022).
AOSD	In florid lesions, apoptotic keratinocytes especially located in the upper layer of the epidermis can be observed. The Upper to mid-dermis shows a perivascular infiltrate consisting of lymphocytes and neutrophils (Lee et al., 2005).
SchS	Variable dense dermal infiltrate consisting of neutrophils arranged alongside collagen bundles without evidence of vasculitis (Lipsker, 2010).
PFAPA	No reports on histopathological findings.

Abbreviations: AOSD, adult-onset Still disease; CANDLE, chronic atypical neutrophilic dermatosis with lipodystrophy and elevated temperature; DIRA, deficiency of IL-1RA; DITRA, deficiency of IL-36RA; PFAPA, periodic fever, aphthous stomatitis, pharyngitis, and adenitis; SAPHO, synovitis, acne, pustulosis, hyperostosis, osteitis syndrome; SAVI, STING-associated vasculopathy with onset in infancy; SchS, Schnitzler syndrome.

The table provides an overview of the histopathological presentation of noninflammasome-mediated autoinflammatory diseases.

### TNF receptor‒associated AIDs

The TNF receptor (TNFR) is an essential player in the innate immune system, regulating inflammation and cell death (Wang and Lin, 2008). The cytokine TNFα mediates essential functions influencing cell proliferation, immune regulation, as well as cell death and survival. These functions are facilitated through the signaling of a family of TNFRs, which, if dysregulated, can lead to a wide variety of undesired symptoms (Lobito et al., 2011). The autoinflammatory TNF-associated disease (TRAPS) is associated with autosomal dominant missense mutations of the *TNFRSF1A* on chromosome 12 (McDermott et al., 1999). *TNFRSF1A* codes for the type I transmembrane protein TNFR1, and over 180 low- and high-penetrance mutations have been reported (Milhavet et al., 2008). Whereas low-penetrance mutations are associated with none or only mild signs of TRAPS, high-penetrance mutations are always disease-causing variants showing a fulminant phenotype (Cantarini et al., 2014; Gaggiano et al., 2020; Lachmann et al., 2014; McDermott et al., 1999; Ruiz-Ortiz et al., 2017). They disrupt structural important disulfide bonds on the protein’s ectodomain, affecting three of the four cysteine-rich domains (CRDs), which are encoded on exons 2‒6 (Banner et al., 1993; UniProt Consortium, 2019). These CRDs are essential for the initial homotrimerization (CRD1) of the receptor and its binding to TNFα (CRD2 and 3) (Rebelo et al., 2006).

**Figure 2:**
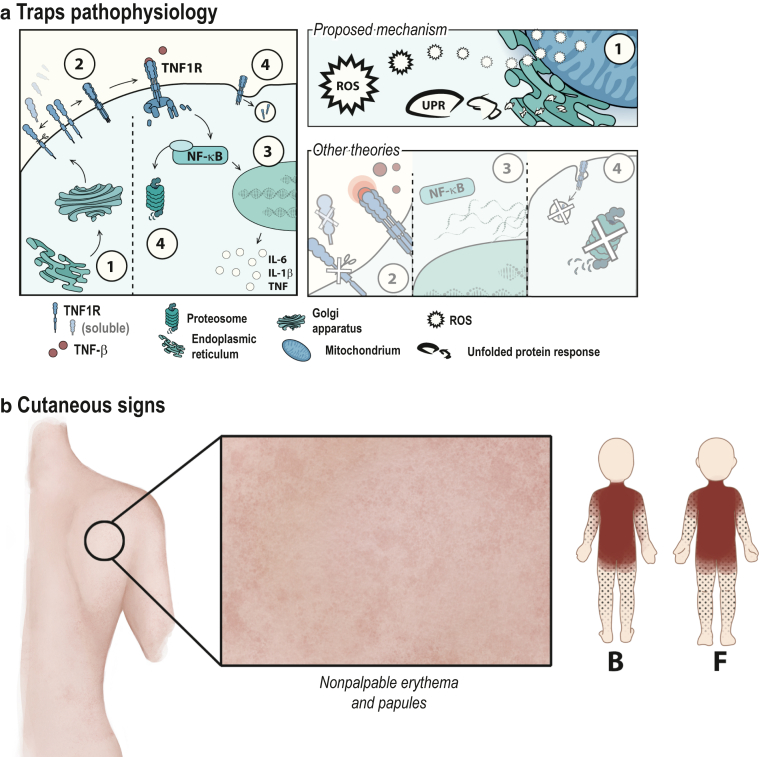
**Pathogenesis of TRAPS and its associated mutations.** (**a**) The lifecycle of the TNFR1 is depicted (left panel) with its associated possible pathogenic disruptions (right panel) through circles 1‒4. After transcription, TNFR1 is translated into the ER and is properly folded (1). TNFR1 is stored in the Golgi apparatus until it is transported toward the cell surface. One possible mechanism of TRAPS includes the misfolding of the protein, leading to ER stress, and the production of ROS and UPR. This theory seems to be involved in most pathogenic mutations, whereas the following mechanism seems to be seen in only some variants. Properly folded TNFR1 reaches the cell surface and is either cleaved by TNF-α‒converting enzyme and other metalloproteases to act as an sTNFR or homotrimerizes to bind TNF-α (2). A defective shedding of TNFR1 as sTNFR1 was seen in some patients with TRAPS. Another theory includes an autoactivation or overactivation of the TNFR1. After binding to TNF-α, TNFR1 associates with further adaptor proteins through lateral mobility to initiate the signaling complex (3). This subsequently leads either to the activation of NF-kB and the production of proinflammatory cytokines or to caspase-induced apoptosis through the death domain of TNFR1. Patients with TRAPS showed elevated microRNA important for the regulation of gene silencing. A heightened stimulation of NF-kB could also be a mechanism of TRAPS. Endocytosis of TNFR1 stops the signaling and downmodulates available TNFR1 on the surface of the cell (4). In TRAPS, not all TNFR1 mutants may be cleared through the proteasome and accumulate in the cytosol, which could lead to ligand-independent signaling processes. (**b**) TRAPS rash is migratory. Erythematous, nonpruritic, tender maculae and papules on the trunk progressively coalesce into patches and plaques and spread toward the limbs. Periorbital edema and less common skin manifestations are not shown. B denotes the back view, and F denotes the front view. ER, endoplasmic reticulum; sTNFR1, soluble TNFR1; TRAPS, TNF receptor‒associated autoinflammatory disease; UPR, unfolded protein response.

TNFR1 is translated into the endoplasmic reticulum (ER) and stored in the Golgi apparatus until it is transported toward the cell surface. It is either cleaved by TNFα-converting enzyme and other metalloproteases to act as a soluble TNFR (sTNFR), regulating the balance of available TNFα, or it homotrimerizes with other TNFR1 (D’Alessio et al., 2012; Porteu and Hieblot, 1994). After homotrimerization, TFNR1 can bind to TNFα and associate with additional adapter proteins through lateral movement on the cell surface to initiate the signaling complex (Morton et al., 2019). This subsequently leads either to the activation of NF-κB and the production of proinflammatory cytokines or to caspase-induced apoptosis through the death domain of TNFR1. Endocytosis of TNFR1 stops the signaling and downmodulates available TNFR1 on the surface of the cell (Figure 2a, left panel) (Jarosz-Griffiths et al., 2019; Lobito et al., 2011). Although the pathophysiology of TRAPS is not yet fully understood, several studies suggest multiple mechanisms leading to the hyperinflammatory state seen in this condition. These theories can be divided into four categories: mechanisms related to (i) faulty regulation and protein folding and (ii) dysregulated actions involving the TNFR1 at the cell surface as well as processes disrupting (iii) the signaling pathway or the (iv) degradation of the protein (Figure 2a, right panel).

Even though nearly all pathogenic mutations influence the protein’s extracellular domain, the pathogenesis proves to be an interwoven net of multiple processes influencing each other with significant variability depending on the variant.

#### Clinical signs and symptoms

From the clinical perspective, TRAPS is characterized by periodic fevers, painful erythematous migratory rashes, and periorbital edema as stipulated by the Eurofever criteria (Gattorno et al., 2019). Arthralgia, myalgia, abdominal pain, and malaise are additionally common findings together with conjunctivitis, pleuritic chest pain, headache, and lymphadenopathy. In 10% of untreated cases, secondary AA amyloidosis is possible (Lachmann et al., 2014; Papa et al., 2021; Toro et al., 2000). Disease onset is usually in childhood with a median age of 4.3 years. In 9.1% of patients, initial symptoms occur after the age of 30 years (Lachmann et al., 2014). The underlying trigger can only be identified in about a third of flare ups. Common triggers include (emotional) stress; infection; injury; and hormonal changes, such as the menstrual cycle, whereas pregnancy is thought to be a mitigating factor (Kimberley et al., 2007; Kriegel et al., 2003; Lachmann et al., 2014; Papa et al., 2021). The duration of flares varies from a few days to months, with a typical duration of 2 weeks. Flares usually occur every 6‒12 weeks. Extended periods without flares up are possible (Kimberley et al., 2007; Lachmann et al., 2014; Papa et al., 2021). Laboratory findings during flares include leukocytosis, elevated acute-phase reactants (CRP, serum amyloid A, haptoglobin, fibrinogen), and an increased erythrocyte sedimentation rate (ESR) (Kastner, 2005; Shwin et al., 2017; Toro et al., 2000; Williamson et al., 1982).

#### Cutaneous signs

TRAPS rash is characterized by migratory, erythematous, nonpruritic, tender patches or plaques with underlying myalgias and periorbital edema (Romano et al., 2022; Toro et al., 2000; Williamson et al., 1982). Early signs of a flare include macules and papules on the trunk that progressively coalesce into patches and plaques and spread toward the limbs (Schmaltz et al., 2010). Other manifestations include urticaria, erysipelas-like rash, small-vessel vasculitis, angioedema, and annular as well as serpiginous plaques (Figure 2b) (Cattalini et al., 2013; Dodé et al., 2002; Jain et al., 2018; Schmaltz et al., 2010; Yao et al., 2012; Zhao et al., 2020).

#### Treatment

The major treatment goals of TRAPS are symptom control during flare ups, reduction of flare frequency, and prevention of secondary AA amyloidosis (Romano et al., 2022). Nonsteroidal anti-inflammatory drugs (NSAIDs) and corticosteroids are used to gain control over symptoms during acute flare ups and are both given on demand (Ter Haar et al., 2015, 2013). However, corticoid-sparing agents are usually required as maintenance therapy. The treatment of choice is IL-1 blockade with canakinumab, which is licensed for TRAPS treatment, with anakinra being a possible alternative (Papa et al., 2021; Romano et al., 2022; Ter Haar et al., 2015, 2013). Owing to its short half-life, anakinra can also be given on demand (Grimwood et al., 2015). Less beneficial treatment options include the IL-6 inhibitors tocilizumab and colchicine (Kuemmerle-Deschner et al., 2020). Etanercept can be helpful in some patients. However, its effects diminish over time. Other TNFα inhibitors, such as infliximab or adalimumab, showed no clear benefit (Kuemmerle-Deschner et al., 2020; Ter Haar et al., 2013).

## IFN-Associated AIDs

IFNs are the base of a complex innate immune response system to battle viral attacks. Type 1 IFNs, especially IFN-α and IFN-β, increase the resistance of cells against the replication of viruses. They also augment the presentation of viral factors for the activation of antigen-presenting cells such as dendritic cells and macrophages and activate NK cells. These mechanisms ensure that banal viral infections are often resolved without influencing the host’s daily life. In patients with interferonopathies, this vital response system is dysregulated, leading to autoinflammation and an abnormal response to viral infections and other stressors.

In CANDLE, the IFN response system is influenced by a failed degradation mechanism caused by the reduction of the proteolytic activity of the proteasome (Figure 1, bottom left) (Torrelo et al., 2010). The steady autoinflammation is easily seen by a characteristic combination of cutaneous lesions involving violaceous plaques, edema of eyelids and lips, as well as progressive lipodystrophy present on the skin since infancy (Torrelo, 2017). In contrast, STING-associated vasculopathy with onset in infancy (SAVI) is induced by a gain-of-function mutation in a viral sensor, leading to the overactivation of the IFN response system (Figure 1, bottom right) (Liu et al., 2014). It shows vasculitic lesions often associated with ulcers and gangrene eventually leading to loss of the acra. Although many more IFN-related AIDs are known, these two mechanistically different diseases CANDLE and SAVI present with memorable cutaneous signs starting in early infancy.

### CANDLE

The rapid response to a viral threat leads to the production of proinflammatory cytokines, chemokines, other proteins, and small molecules such as ROS or nitrous oxide (NOS) to aid the overall microbiocidal activity. Although these factors help to eliminate the intruder or inform neighboring cells of the threat, they may have profound consequences for the cell itself. Especially, ROS and NOS may irreversibly damage proteins inside the cell (Johnston-Carey et al., 2015). These damaged proteins need to be cleared in a coordinated manner to reduce stress and ensure the cell’s survival. In homeostasis, waste proteins get degraded by the proteasome after ubiquitination. In inflammation, the constitutively active proteasome gets help from the immune proteasome (Ciechanover, 2012; Ebstein et al., 2012; Johnston-Carey et al., 2015; Kasahara and Flajnik, 2019). This immunoproteasome is mainly induced in hematopoietic cells through type I IFNs and helps the cell to meet the elevated demand for degrading waste proteins in a state of inflammation (Aki et al., 1994; Ebstein et al., 2012). It also heightens the degradation of pathogen-related proteins to present them on the cell’s surface through major histocompatibility complex I (Ebstein et al., 2012). The building blocks of proteosomes consist of a subunit specialized in recognizing ubiquitinated proteins transporting it to a core subunit specialized in proteolytic degradation (Baumeister et al., 1998; Johnston-Carey et al., 2015). In CANDLE, a proteosome-associated autoinflammatory disease (PRAAS), mutations of genes involved in the assembly or the proteasome’s function lead to the loss of the ability to degrade waste (Figure 1, bottom left) (Torrelo, 2017). The first mutation associated with CANDLE was detected nearly 10 years ago in the gene *PSMB8* (Liu et al., 2012). It adds a crucial chymotrypsin-like activity to the core subunit and might also play a role in assembling the immunoproteasome (Johnston-Carey et al., 2015; Torrelo, 2017). Various other variants of genes such as *PSMB4*, *PSMB9*, *PSMA3*, and the recently discovered loss-of-function mutation in *PSMB10* (International Society of Systemic Auto-Inflammatory Diseases, 2019) as well as *PSMG2* (de Jesus et al., 2019) affect either the immunoproteasome directly or general parts of the proteasome subunits or its assembly. A recent study (Davidson et al., 2022) identified protein kinase R (PKR) as a sensor recognizing proteotoxic stress because of the accumulation of IL-24 in a human cell line model. IL-24 is constitutively secreted by the ER and might act as a control mechanism for a functional proteasome degradation system. Patients with PRAAS exhibited increased levels of phosphorylated PKR and IL24 as well as smaller isoforms of *IL24* mRNA (Davidson et al., 2022). The highly reduced function of the degradation of waste products further induces proinflammatory reactions leading to a vicious cycle and an overall heightened type I IFN production even after trivial viral infections and other stressors (Brehm et al., 2015; Ebstein et al., 2012; McCarthy and Weinberg, 2015). Plasmacytoid dendritic cells produce high amounts of type I IFN in cutaneous lesions of patients with CANDLE (Jegalian et al., 2009; Torrelo et al., 2015). Type I IFN mRNA can be used as a biomarker in patients with CANDLE, and the inhibitors directly targeting the IFN-induced Jak‒signal transducer and activator of transcription (STAT) pathway seem to ameliorate symptoms (Sanchez et al., 2018).

#### Clinical signs and symptoms

Clinically, CANDLE is an AID characterized by a combination of symptoms with multiorgan involvement (Arima et al., 2011; Brehm et al., 2015; de Jesus et al., 2019; International Society of Systemic Auto-Inflammatory Diseases, 2019; Liu et al., 2012). The true prevalence of this rare AID is still unknown, with more than 45 cases found in the medical literature (Torrelo, 2017). The first signs of disease usually occur within the neonatal period, whereas disease onset at higher age is rare but possible (Cetin Gedik et al., 2022; Torrelo et al., 2010). Patients present with almost daily recurring fevers, pruritic rashes, and edema of the lips and eyelids. Stress, cold temperatures, or viral infections are possible triggers for fever attacks and cutaneous lesions (Torrelo, 2017). During the further course of the disease, patients develop eponymous lipodystrophy. Failure to thrive commonly occurs; however, the presence of developmental delay is rare (Cetin Gedik et al., 2022). Other disease symptoms include arthralgias, clubbing of the fingers and toes, myositis, chronic chondritis leading to ear and saddle-nose deformities, conjunctivitis, nodular episcleritis, aseptic meningitis, calcification of the basal ganglia, metabolic syndrome, hepatosplenomegaly with a prominent abdomen, and inflammation of other organs (Brehm et al., 2015; Torrelo et al., 2010). If left untreated, CANDLE results in progressive organ damage eventually leading to early mortality (Cetin Gedik et al., 2022). Laboratory analysis shows hypochromic anemia, leukocytosis, thrombocytosis, increased ESR, and increased levels of CRP. Occasionally, liver enzymes can be elevated (Brehm et al., 2015; Torrelo et al., 2010). An elevated peripheral blood IFN signature is also a typical but not disease-specific sign of CANDLE (Cetin Gedik et al., 2022).

#### Cutaneous signs

**Figure 3:**
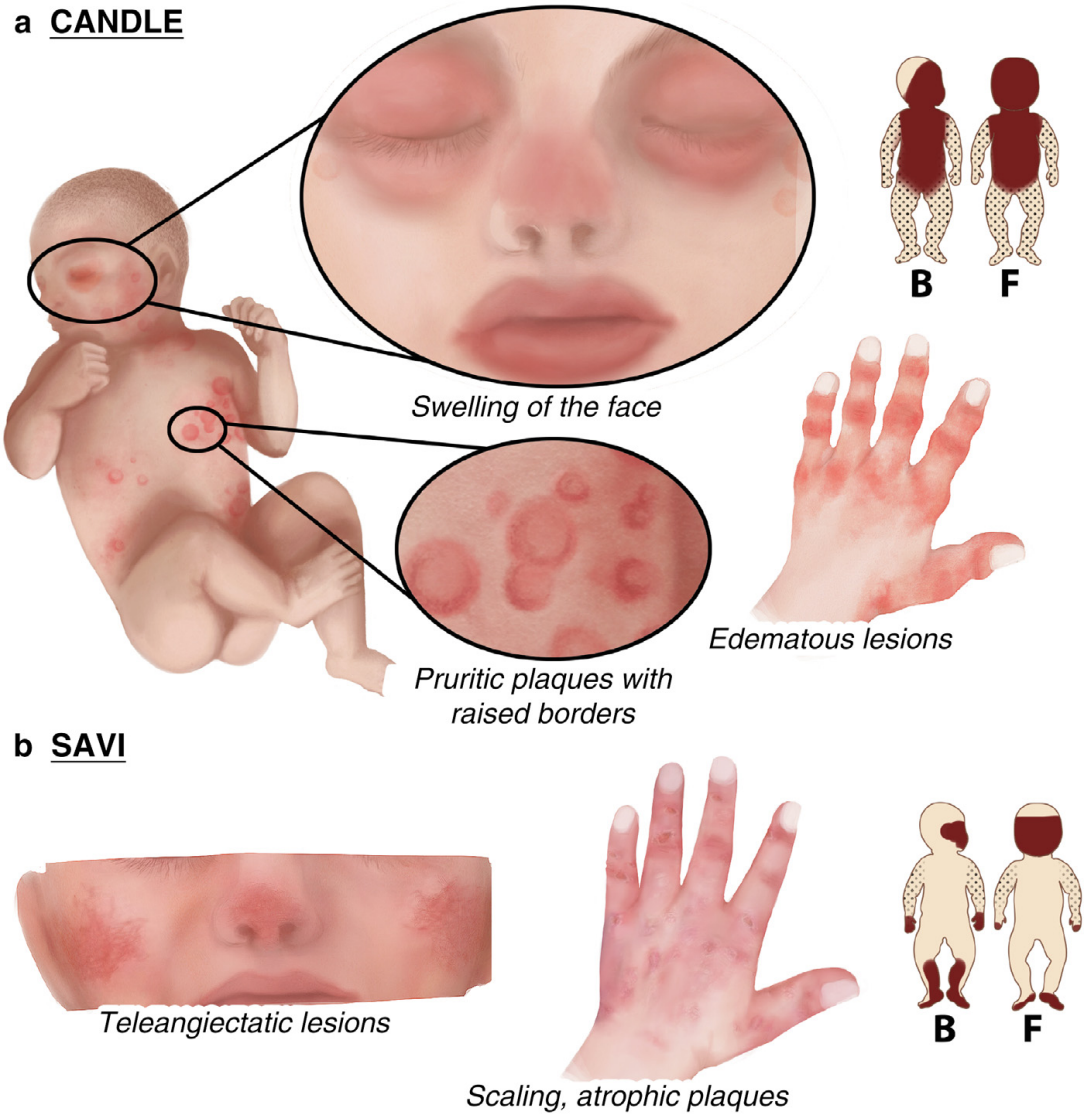
**Cutaneous signs of IFN-associated AIDs.** (**a**) CANDLE: a unique combination of dermatologic findings: perniotic, edematous lesions on acra in the first few months of life; later flat, erythematous or violaceous plaques with raised borders (diameter 1‒5 cm) on the face, trunk, and extremities; persistent, erythematous to violaceous swellings of the lips and eyelids; lipodystrophy starting at the face and progressing to the trunk and limbs. (**b**) SAVI: erythematous to violaceous, infiltrated, ulcerating plaques on the acra, dorsum of the hands, thighs, and soles; facial erythema resembling malar rash; and telangiectatic lesions on the cheeks, nose, and extremities. Other findings (e.g., gangrene, saddle-nose deformity) are not shown. B denotes the back view, and F denotes the front view. AID, autoinflammatory disease; CANDLE, chronic atypical neutrophilic dermatosis with lipodystrophy and elevated temperature; SAVI, STING-associated vasculopathy with onset in infancy.

The combination of dermatologic findings in CANDLE is unique. In the first few months of life, patients present with perniotic, red-to-violaceus, edematous lesions occurring on the acral digits with cold exposure as a possible trigger (Arima et al., 2011; Torrelo, 2017). During infancy or childhood, annular, flat, erythematous or violaceous, pruritic plaques with raised borders ranging from 1 to 5 cm in diameter start to develop. They are usually located on the face and trunk but may also involve the extremities, including hands, palms, feet, and soles. New lesions develop weekly or every few weeks and gradually resolve over the course of a few weeks, occasionally resulting in hyperpigmentation. In addition, patients develop persistent, erythematous to violaceous swellings of the lips and eyelids (Figure 3a) (Arima et al., 2011; Brehm et al., 2015; Torrelo et al., 2010). Eczema and flare-related urticaria have been reported to occur as well (Schwartz et al., 2021). Lipodystrophy has its onset in early childhood, with progressive subcutaneous fat loss starting at the face and progressing to the trunk and limbs with the upper typically more affected than the lower extremities (Brehm et al., 2015; Torrelo et al., 2010).

#### Treatment

Treatment focuses on controlling disease activity to prevent organ damage or its progression. However, optimal treatment of CANDLE remains challenging with both methotrexate and corticosteroids leading to a partial improvement of fever attacks and cutaneous lesions. In some cases, NSAIDs can be used to control fever attacks (Torrelo et al., 2010). Jak1/2 inhibitors represent a promising new treatment approach with improved efficacy compared with IL-1 inhibitors, TNFα inhibitors, colchicine, azathioprine, ciclosporin, dapsone, or intravenous Ig (Cetin Gedik et al., 2022; Torrelo et al., 2010). In a case report, treatment with Jak1/2 inhibitor baricitinib led to sufficient disease control because fever attacks stopped, skin rashes improved, periorbital swelling disappeared, musculoskeletal symptoms resolved, and growth velocity increased (Boyadzhiev et al., 2019). In a case series, Jak1/2 inhibitor baricitinib led to improved disease control with remission occurring in 50% of patients (Sanchez et al., 2018).

### SAVI

There are numerous sensor and adapter proteins that help cells in combating invading viruses. STING is one of them and was found to acquire a gain-of-function mutation in patients with SAVI. STING is anchored at the membrane of the ER and gets indirectly activated by viral DNA and abnormal DNA derived from bacteria or damaged cells (Liu et al., 2021; Sun et al., 2009). After activation, STING travels to the Golgi complex and the perinuclear compartment, where it further interacts with several proteins, ultimately leading to the translocation of the IRF3 to the nucleus and the induction of type I IFNs (Figure 1, bottom right) (Tsuchiya et al., 2016; Zhao et al., 2016). Although the activation mechanism between DNA and RNA viruses varies (Liu et al., 2021), SAVI-associated mutations of *TMEM173*, the gene coding for STING, lead to a constitutional activated STING protein and subsequently a heightened production of IFN-β and autoinflammation similar to that in other interferonopathies (Ergun et al., 2019). Liu et al. (2014) showed the constitutive activation of the STING pathway in peripheral blood monocytes and the upregulation of the reactivity to stimulating factors in fibroblast of patients with SAVI. In these fibroblasts, the Jak1/2 inhibitor baricitinib inhibiting the IFN pathway shows promising results (Kim et al., 2018; Liu et al., 2014; Sanchez et al., 2018).

#### Clinical signs and symptoms

As the name suggests, SAVI is an AID with onset in early infancy with a median age of symptom onset <6 months. Only 1 of 60 reported cases had adulthood disease onset (Frémond et al., 2021). The exact prevalence of this rare AID is unknown (Frémond et al., 2021). SAVI is characterized by small-vessel vasculitis with recurrent cutaneous rashes and sometimes cartilage damage reflected as ear and/or saddle-nose deformities; interstitial lung disease with dyspnea, tachypnea and/or, cough eventually leading to respiratory failure; systemic inflammation; recurrent fevers; failure to thrive, and arthritis or arthralgia (Frémond et al., 2021; Liu et al., 2014). Rarely, neuroimaging shows basal ganglia calcifications (Cetin Gedik et al., 2022). CRP levels and ESR are usually increased (Liu et al., 2014). The peripheral blood IFN signature is also elevated, similar to that in CANDLE (Cetin Gedik et al., 2022). In addition, >50% of patients with SAVI are positive for antinuclear antibodies (35 of 56 patients), antineutrophil cytoplasmic antibodies (ANCAs) (15 of 21 patients) (perinuclear ANCA and cytoplasmic ANCA), and rheumatoid factor (17 of 30 patients) (Frémond et al., 2021). Leukopenia and thrombocytosis occur frequently (Liu et al., 2014). Levels of IgG and IgA are elevated, and levels of IgM and complement are shown to be within the reference range (Liu et al., 2014).

#### Cutaneous signs

Skin lesions can be present at disease onset or develop months afterward (Tang et al., 2021). As shown in a large case series published by Frémond et al. (2021), 86% of patients exhibit cutaneous lesions. Patients usually present with erythematous to violaceous, infiltrated plaques on the digits and nose and ears, dorsum of the hands, thighs, and soles (Figure 3b). Plaques ulcerate in most of the patients. Patients also exhibit facial erythema resembling malar rash and telangiectatic lesions on the cheeks, nose, and extremities. In addition, nailfold capillary tortuosity, alopecia, livedo, urticarial lesions on the upper extremity, oral aphthosis, and gingivostomatitis have been described (Frémond et al., 2021; Schwartz et al., 2021; Tang et al., 2021). In severe cases (19% of patients), SAVI leads to extensive tissue loss because of vascular occlusion resulting in ear deformity, nasal septum perforation, saddle-nose deformity, loss of nails, and gangrene and consecutive loss of fingers or toes (Frémond et al., 2021; Liu et al., 2014).

#### Treatment

As seen in CANDLE, inhibition of the Jak/STAT pathway through Jak inhibitors is a promising treatment option, whereas treatment with immunomodulators such as glucocorticoid monotherapy, conventional synthetics (e.g., methotrexate, leflunomide), or biologic disease-modifying drugs (e.g., etanercept, infliximab, anakinra, belimumab, rituximab, tocilizumab), hydroxychloroquine, mycophenolate motefil, cyclophosphamide, intravenous Ig, colchicine, or thalidomide led to no or incomplete response (Cetin Gedik et al., 2022; Liu et al., 2014). Treatment with Jak inhibitors such as ruloxitinib and baricitinib, initially in combination with systemic corticosteroids, allowed for a reduction of disease severity (Frémond et al., 2021; Sanchez et al., 2018). Jak inhibitor tofacitinib was associated with unsatisfactory treatment response (Tang et al., 2020).

## IL-1 Family Receptor Antagonist‒Associated AIDs

The IL-1 family includes many cytokines and receptors facilitating the response against harmful pathogens. One of the most prominent agents is IL-1β. As seen in, for example, inflammasome-mediated AIDs, the massive IL-1β release can lead to the destruction of the surrounding tissues and needs to be tightly controlled. The IL-1RA prevents a vicious cycle of autocrine stimulation between IL-1β and the IL-1 receptor (Lennard, 2017). When this mechanism fails, a chronic inflammatory state leads to a rare, life-threatening disease called deficiency of IL-1RA (DIRA) (Aksentijevich et al., 2009). Because the IL-1 family spans a wide range of cytokines with a similar regulatory mechanism, DIRA is not the only AID known to be the outcome of a failed receptor antagonist. DITRA describes patients with a defect in the IL-36RA (Marrakchi et al., 2011).

### DIRA

Binding of IL-1β to the IL-1 receptor leads to heterodimerization and the activation of a potent proinflammatory signal cascade (Fields et al., 2019; Wang et al., 2010), priming the cell for further production of pro‒IL-1β (Avolio et al., 2014). Because this can lead to a circle of autocrine stimulation, cells that produce IL-1β (in particular, monocytes and macrophages, neutrophils, dendritic cells, and epithelial cells) also code the antagonistic partner IL-1RA (Eisenberg et al., 1991). IL-1RA binds with high affinity to the IL-1 receptor without inducing the conformational change needed for proinflammatory signaling (Eisenberg et al., 1991; Fields et al., 2019). This way, IL-1RA competitively inhibits the activation of the IL-1 receptor and lays the groundwork for a controlled inflammatory response. Patients with DIRA show missense or nonsense mutation in the gene *IL1RN*, which codes for IL-1RA (Aksentijevich et al., 2009). More than 15 mutations with varying pathogenic potentials are registered at InFevers, and sequencing efforts continuously uncover new variants (Milhavet et al., 2008). A recent study reported a new mutation leading to DIRA in Brazil (Mendonça et al., 2020), after additions from Turkey (Sözeri et al., 2018) and India (Mendonca et al., 2017) in 2018 and 2017, respectively. As far as reported, these mutations lead to a faulty protein expression or to no production of the protein at all (Figure 1, top right) (Aksentijevich et al., 2009). Monocytes affected with DIRA-associated mutations showed elevated production of several proinflammatory cytokines, including IL-1, IL-6, TNF, and MIP-1α (Aksentijevich et al., 2009; Reddy et al., 2009). IL-6 is a typical proinflammatory cytokine heightening the production of acute-phase proteins as well as inducing neutrophilia and T helper 17 (Th17) T-cell differentiation, both seen in the inflamed skin of patients affected by DIRA (Aksentijevich et al., 2009; Naseem et al., 2016; Suwa et al., 2000). The impact of IL-1RA to regulate inflammatory responses can be nicely shown through the effect of recombinant IL-1RA because the response to the treatment stops with discontinuation of the therapy.

#### Clinical signs and symptoms

DIRA is a rare AID of unknown global prevalence but was estimated to occur in 1 case per 6,300 inhabitants in Puerto Rico (Aksentijevich et al., 2009). The disease usually presents at birth or during the neonatal period and is typically characterized by chronic inflammation with occasionally occurring flare ups (Aksentijevich et al., 2009). Clinical features include a pustular skin rash triggered by mechanical stress, nail changes, oral mucosal lesions, sterile osteomyelitis, periostitis, balloon-like widening of bones, swollen joints, and severe bone pain. Hepatosplenomegaly and abdominal distention with a caput medusae are other possible signs of the disease. However, fever is uncommon (Romano et al., 2022). DIRA is usually fatal if left untreated. Laboratory studies show elevated levels of acute-phase reactants, leukocytosis, thrombocytosis, and anemia (Aksentijevich et al., 2009; Brau-Javier et al., 2012; Reddy et al., 2009).

#### Cutaneous signs

**Figure 4:**
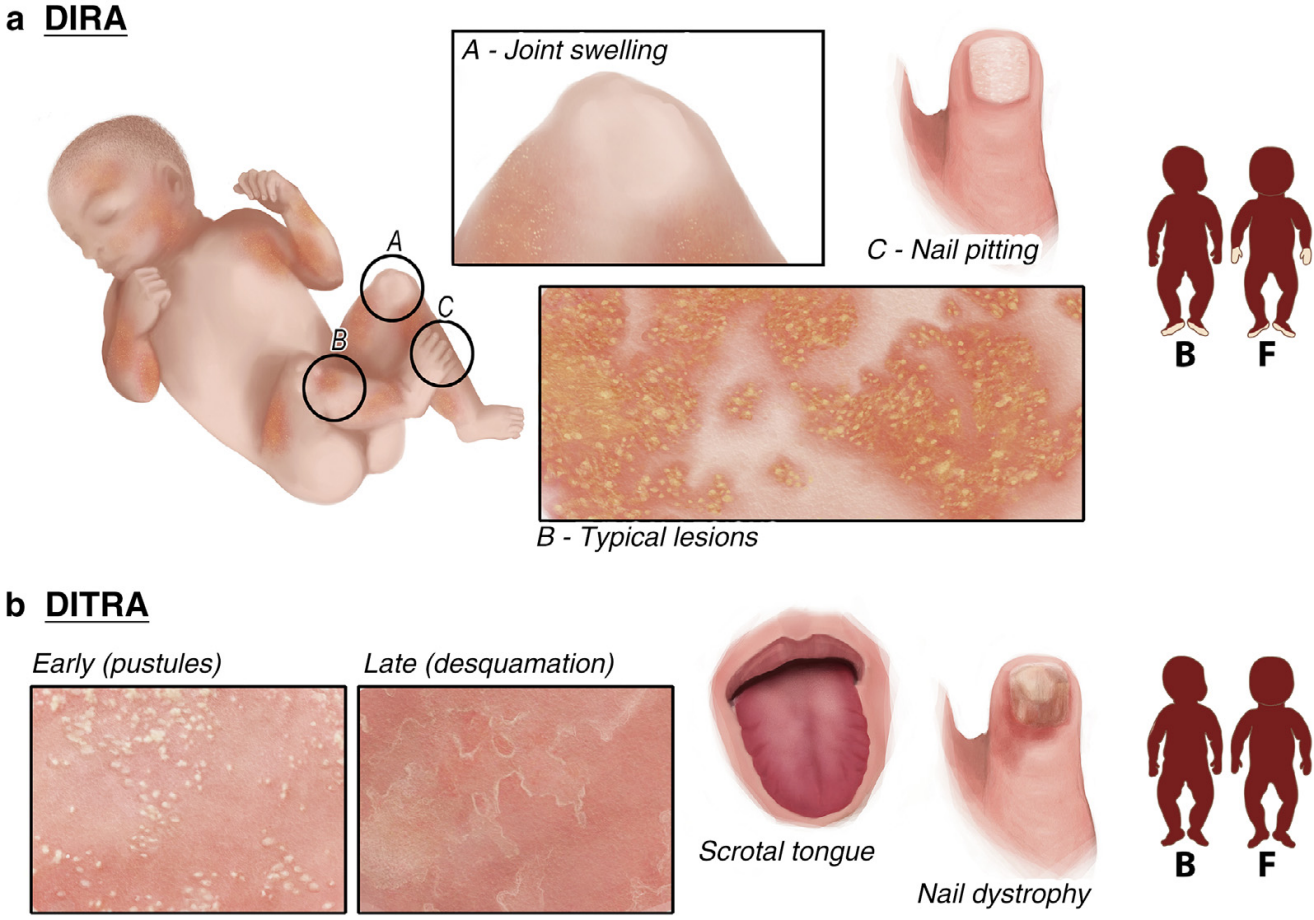
**Cutaneous signs of DIRA and DITRA.** (**a**) DIRA: erythematous plaques studded with sterile follicular pustules limited to one area or with generalized location and nail involvement (pitting). Other findings (e.g., ichthyosiform lesions, stomatitis) were not shown. (**b**) DITRA: generalized erythematous plaques studded with sterile pustules with consecutive desquamation, scrotal tongue, and nail dystrophy. Benign migratory glossitis is not shown. B denotes the back view, and F denotes the front view. DIRA, deficiency of IL-1RA; DITRA, deficiency of IL-36RA.

Cutaneous manifestations can either be mild with erythematous plaques and sterile follicular pustules limited to one area or severe with generalized pustulosis or ichthyosiform lesions. Sparing of the palms and soles has been reported. A positive skin pathergy test, oral mucosal lesions (ulcers, stomatitis), and nail involvement (pitting, onychomadesis) are possible findings (Figure 4a) (Aksentijevich et al., 2009; Brau-Javier et al., 2012; Goldbach-Mansky, 2012; Minkis et al., 2012; Reddy et al., 2009; Romano et al., 2022).

#### Treatment

Inhibition of IL-1α appears to be important in stopping bone inflammation. Hence, anakinra and rilonacept are the mainstays of treatment because they block both IL-1α and IL-1β as opposed to canakinumab, which only inhibits IL-1β (Romano et al., 2022). As shown in a report on the basis of the Eurofever Registry, anakinra induced complete remission in the majority of patients (Ter Haar et al., 2013). Canakinumab has been used successfully in one patient, whereas another patient continued to experience flare ups while on therapy (Mendonça et al., 2020; Ulusoy et al., 2015). Disease-modifying antirheumatic drugs and corticosteroids appear to be only partially effective (Aksentijevich et al., 2009).

### DITRA

Similar to DIRA, DITRA involves the regulation of the proinflammatory effect of an IL-1 family cytokine through its receptor antagonist. DITRA shows a mutation in the gene *IL36RN*, which codes for IL-36RA (Figure 1, top left) (Aksentijevich et al., 2009; Marrakchi et al., 2011; Onoufriadis et al., 2011). Mutations in *IL36RN* are not specific for DITRA because they can also lead to palmoplantar pustulosis and acrodermatitis continua of Hallopeau, both being pustular entities related to psoriasis (Setta-Kaffetzi et al., 2013). The three different IL-36 (α, β, and γ) are lesser known members of the IL-1 family and are, similar to their antagonist IL-36RA, primarily expressed in epithelial tissues of barrier sites such as the skin (Bassoy et al., 2018; Zhou and Todorovic, 2021). A general proinflammatory state could be observed in DITRA because monocytes from patients showed a heightened production of IL-1β, IL-1α, IL-6, IL-8, and TNF similar to monocytes from patients with DIRA (Aksentijevich et al., 2009; Marrakchi et al., 2011; Onoufriadis et al., 2011). IL-36, which was shown to be highly elevated in skin lesions from patients with DITRA, stimulates keratinocytes and drives the Th17/23 cell axis, which is essential to protect the body’s outer layers against pathogens. Its high expression in epithelial tissues might be the reason for the more skin-specific hyperinflammation and negligible systemic involvement seen in DITRA compared with those seen in DIRA (Aksentijevich et al., 2009; Guglani and Khader, 2010; Marrakchi et al., 2011; Zhou and Todorovic, 2021).

#### Clinical signs and symptoms

DITRA presents with recurrent flares of generalized erythematous patches studded with pustules (pustular psoriasis), high bouts of fever, and systemic inflammation (Marrakchi et al., 2011; Onoufriadis et al., 2011). Malaise and organ involvement in terms of hepatosplenomegaly with ascites and pericardial and pleural effusion can also occur (Ellingford et al., 2016; Körber et al., 2013; Marrakchi et al., 2011). The prevalence of generalized pustular psoriasis is estimated to be two per one million persons (Körber et al., 2013). The exact prevalence of DITRA is unknown, with about 200 cases being reported in the medical literature (Hospach et al., 2019). Mössner et al. (2018) detected *IL36RN* mutations in 20 of 61 (33%) investigated patients with generalized pustular psoriasis. The disease onset is varying, ranging from an age of 1 week to age of 72 years, with a mean age of 33.4 ± 22.4 years (Körber et al., 2013; Marrakchi et al., 2011; Mössner et al., 2018). The course of the disease is usually episodic, less often continuous (Mössner et al., 2018). A recent study reported the flare duration of a few days to a week (Salik et al., 2021). In cases of episodic DITRA, flare ups occur at irregular time intervals (Mössner et al., 2018). Disease flares can be triggered by viral or bacterial infections (e.g., urinary tract infection; gastroenteritis; upper respiratory tract infections such as bronchitis, common cold, and sinusitis), stress, drug intake (penicillin, sulfonamides, codeine, paracetamol, metamizole, acetylsalicylic acid, verapamil), withdrawal of drugs (retinoid, methotrexate, corticosteroids), menstruation, pregnancy, red wine, and surgical interventions (Körber et al., 2013; Marrakchi et al., 2011; Mössner et al., 2018; Onoufriadis et al., 2011). Blood tests show elevated levels of CRP, increased ESR, and leukocytosis with neutrophilia (Marrakchi et al., 2011; Onoufriadis et al., 2011).

#### Cutaneous signs

Patients present with generalized erythematous plaques studded with sterile pustules. In most patients, skin manifestations occur episodically with a sudden onset and subsequent desquamation (Marrakchi et al., 2011). Involvement of the tongue (benign migratory glossitis, scrotal tongue) and nail dystrophy can additionally occur (Figure 4b) (Körber et al., 2013; Marrakchi et al., 2011).

#### Treatment

Treatment options include acitretin; corticosteroids; methotrexate; ciclosporin; and TNFα, IL-1, IL-12/23, and IL-17 inhibitors (Hospach et al., 2019; Onoufriadis et al., 2011). Acitretin with or without concomitant methotrexate has been used successfully in a few published cases (Ellingford et al., 2016; Onoufriadis et al., 2011). There is also evidence for the beneficial effect of corticosteroids and ciclosporin (Onoufriadis et al., 2011). Treatment with TNF-α inhibitors induced a complete remission in 7 of 12 flares (58%). IL-1, IL-12/23, and IL-17 led to a complete remission of one of eight (13%), four of four (100%), and four of four (100%) flares, respectively (Hospach et al., 2019). Targeted therapy inhibiting the IL-36 pathway might be a promising treatment in the future (Bachelez et al., 2019).

## Polygenic Autoinflammatory Syndromes

Polygenic AIDs are defined by an overactive cytokine production of the innate immune system with no underlying monogenic driver. In many cases, infectious triggers or mutations predisposing to the disease are being discussed, but owing to the multifactorial influence, consistent pathogenesis is often missing. Synovitis, acne, pustulosis, hyperostosis, osteitis syndrome (SAPHO); periodic fever, aphthous stomatitis, pharyngitis and adenitis (PFAPA), SchS, and adult-onset Still disease (AOSD) represent some of these polygenic AIDs showing a wide variety of cutaneous signs.

### SAPHO

SAPHO is a complex inflammatory disease with predominantly autoinflammatory characteristics bearing additional autoimmune features and signs of infectious disease. No clear genetic background has been described, but reported familial aggregation (Dumolard et al., 1999; Ferguson et al., 2008) and association with certain SNVs (Assmann et al., 2010; Xu et al., 2019) indicate an underlying genetic susceptibility for the disease. High levels of proinflammatory cytokines strongly stress the autoinflammatory component, but differences in cytokine expression between patients and studies complicate the description of coherent pathogenesis. Elevated IL-6, IL-8, and IL-17 as well as low TNFβ serum levels were observed in SAPHO (Hurtado-Nedelec et al., 2008; Przepiera-Będzak et al., 2016, 2015; Zhang et al., 2019). Zhang et al. (2019) correlated IL-6, IL-8, as well as the IL-17/TNFα quotient to clinical scores measuring disease activity (Visual Analog Scale [VAS] and Bath Ankylosing Spondylitis Activity Index [BASDAI]). The elevation of TNF-α observed in bone biopsies and high levels of *Propionibacterium acnes* were reported to influence the manifestation of the disease (Assmann and Simon, 2011; Gupta et al., 2021; Wagner et al., 2002). *P. acnes*, a Gram-positive commensal skin bacterium (Brüggemann et al., 2004), is found in nearly 50% of bone biopsies, and treatment of colonized skin lesions with antibiotics showed improvement in some patients with SAPHO, suggesting that *P. acnes* might be an infectious trigger of the disease (Assmann et al., 2009; Assmann and Simon, 2011; Berthelot et al., 2018). The effect of antibiotic treatment was often lost after discontinuation (Assmann et al., 2010; Hurtado-Nedelec et al., 2008). Some phylotypes of *P. acnes* were associated with high IL-1β production (Berthelot et al., 2018), which is a known proinflammatory cytokine and a driving force in many other AIDs (Liao et al., 2015). Although heightened IL-1β release after stimulation was indeed observed in a patient with SAPHO (Colina et al., 2010), there was no difference in the IL-1 plasma serum levels compared with that in the healthy controls (Zhang et al., 2019). Interestingly, IL-1 inhibition showed significant alleviation of musculoskeletal manifestations but no change in the severity of skin lesions (Daoussis et al., 2019). The impact of IL-1 and *P. acnes* on SAPHO remains largely obscure and demands further studies.

Other cytokines provide a more coherent but not complete insight into the disease. IL-6 is a classical proinflammatory cytokine known for its bone resorbing characteristics because it stimulates osteoclastogenesis by influencing osteoblast’s expression of RANKL, which was elevated in patients with a high disease activity score (VAS/BASDAI ≥ 4) (Tamura et al., 1993; Wu et al., 2017). RANKL is a key player in bone metabolism, which can also influence the immune system (e.g., in skin inflammation or thymus development) (Ono et al., 2020). Abundant TNF-α and RANKL were observed at sites of inflammatory bone erosions, with TNF-α further stimulating RANKL-induced osteoclastogenesis (Zhang et al., 2001). Zhang et al. (2019) suspected that these processes are highly involved in the generation of bone lesions seen in SAPHO, in accordance with the aggressive bone erosions seen in psoriatic arthritis (Ritchlin et al., 2003). TNF blockers showed promising response rates for musculoskeletal and skin manifestation in SAPHO (Daoussis et al., 2019). The key cytokine connecting innate immunity with adaptive immunity in SAPHO seems to be IL-17. IL-17 is known to connect T cells to neutrophil activation, and its pro-osteoclastogenic properties contribute to the pathogenesis of many rheumatic diseases (Miossec, 2017; Zenobia and Hajishengallis, 2015). IL-17‒producing T cells (Th17 cells) are induced by IL-6 and IL-1β and were shown to be elevated in the serum of patients with SAPHO (Acosta-Rodriguez et al., 2007; Firinu et al., 2014). Recently, Daoussis et al. (2019) connected IL-17 to all hallmark symptoms of the disease: synovitis, acne, pustulosis, hyperostosis, and osteitis. Biopsies of patients with acne and palmoplantar pustulosis showed high levels of IL-17 (Daoussis et al., 2019). IL-17 also has a great impact on neutrophil migration and, together with IL-8, an early-phase chemokine, which strongly attracts neutrophils and to a lesser amount T cells and basophils, could explain the increased infiltration of neutrophils seen in skin biopsies of patients with SAPHO (Acosta-Rodriguez et al., 2007; DeForge et al., 1993; Ferguson et al., 2008). In addition, RNA sequencing of neutrophils revealed an enhancement of neutrophil migration and adhesion markers in SAPHO (Sun et al., 2019). IL-17 inhibitors showed varying response rates with a greater impact on skin manifestations than on musculoskeletal symptoms. It is suspected that the diversity in the efficacy of IL-17 inhibitors could be dependent on the Th17 count (Assmann et al., 2017). More data are needed to evaluate the effectiveness of these newer biologics targeting the IL-17/IL23 axis (Daoussis et al., 2019).

#### Clinical signs and symptoms

SAPHO is a rare AID with an estimated prevalence of 100‒400 cases per million (Huhn et al., 2019). It is characterized by cutaneous and osseoarticular manifestations. A total of 54% of skin manifestations occur before involvement of bones and joints (Li et al., 2016). Dermatologic manifestations include palmoplantar pustulosis, severe acne, and psoriasis vulgaris. The type of cutaneous manifestation depends on various epidemiological factors. Patients with severe acne are usually younger at the onset, with a median age of 20 years, and are predominantly male. Patients with palmoplantar pustulosis are usually female with a median disease onset of 37 years (Li et al., 2020). Clinical features of articular involvement include joint pain; tenderness; swelling; as well as occasional erythema because of hyperostosis, osteitis, and arthritis (Huhn et al., 2019). Nearly all patients with SAPHO suffer from anterior chest wall pain. Radiologic evaluation shows abnormalities, such as osteolysis, sclerosis, and hyperostosis, in 90% of patients. Other common sites of involvement include the lumbosacral regions and peripheral joints (Li et al., 2016). In laboratory analysis, ESR and CRP levels are increased in more than half of the patients (Li et al., 2016).

#### Cutaneous signs

**Figure 5:**
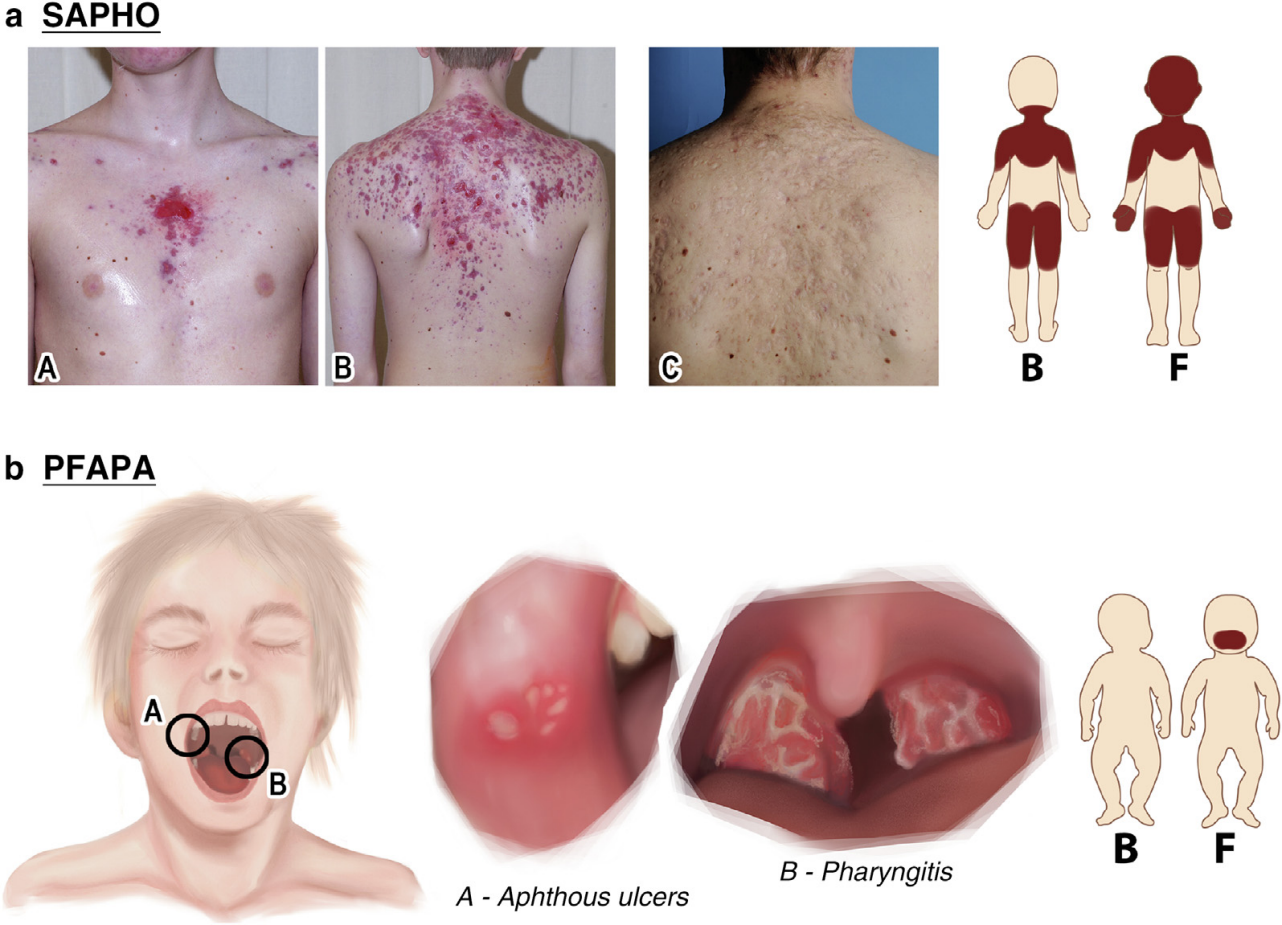
**Cutaneous signs of SAPHO and PFAPA.** (**a**) SAPHO: acne fulminans on the upper trunk (A and B) healing with scarring (C). Hidradenitis suppurativa, psoriasis vulgaris, and palmoplantar psoriasis are not shown. (**b**) PFAPA: small oral aphthae surrounded by erythema on the nonkeratinized oral (A) and pharyngitis (B). Skin rash is not shown. The patient gave consent to the publication of his photographs. B denotes the back view, and F denotes the front view. PFAPA, periodic fever, aphthous stomatitis, pharyngitis, and adenitis; SAPHO, synovitis, acne, pustulosis, hyperostosis, osteitis syndrome.

Skin manifestations of SAPHO include mainly severe acne and/or palmoplantar pustulosis, with psoriasis vulgaris rarely occurring as well (Li et al., 2016). About 70% of patients suffer from palmoplantar pustulosis only, and 7% exhibit acne lesions only. The remainder manifests with combinations of palmoplantar pustulosis, acne, and/or psoriasis vulgaris (Li et al., 2016). Severe acne can present as acne conglobata, acne fulminans, and/or hidradenitis suppurativa. Acne conglobata is characterized by cystic lesions, with interconnecting sinuses and scarring occurring on the face, neck, upper trunk, upper arms, thighs, and buttock (Figure 5a). Acne fulminans refers to a highly inflammatory form of acne with ulcerative lesions. Hidradenitis suppurativa manifests as inflammatory nodules, cysts, abscesses, and sinus tracts usually in the axillary, anogenital, and/or groin area (Kahn and Khan, 1994). Acne occurs predominantly in male patients with SAPHO (Li et al., 2020). Palmoplantar pustulosis is characterized by an abrupt eruption of multiple sterile pustules typically 2‒4 mm in diameter occurring on the palms and soles. Occasionally, lesions can be found on the dorsal aspect of the hands and feet as well (Kahn and Khan, 1994). Palmoplantar pustulosis occurs mainly in female patients with SAPHO syndrome (Li et al., 2020).

#### Treatment

The choice of a therapeutic agent is based on the disease manifestations. Patients with predominant joint involvement are initially treated with NSAIDs or corticosteroids (Colina et al., 2009; Hayem et al., 1999; Li et al., 2016). Patients who do not respond to the initial treatment can be switched to methotrexate, followed by TNF-α inhibitors alone or in combination with methotrexate (Daoussis et al., 2019; Li et al., 2016). Less commonly used treatments are bisphosphonates; phosphodiesterase 4 inhibitor apremilast; and inhibitors of IL-1, IL-17, and IL-12/-23 (Aljuhani et al., 2015; Colina et al., 2009; Daoussis et al., 2019; Wendling et al., 2012, 2017). SAPHO-related acne can be treated with oral antibiotics such as azithromycin and doxycycline (Aljuhani et al., 2015) or isotretinoin (Galadari et al., 2009).

### PFAPA

Pathogenesis of PFAPA is broadly based on an overactivated innate immune system dysregulating T-cell activity. Various hypotheses for the pathogenesis of PFAPA were postulated in the last decade, but none could be proven yet. Neither a monogenic background nor an infectious trigger could be identified. A recent study (Yildiz et al., 2021a) proposed that the disease onset or attack duration might be influenced by *MEFV* gene variants. *MEFV*, the gene responsible for the monogenic AID FMF, and several other genes associated with inflammasomopathies or the activation of the inflammasome (e.g., *ALPK1* [Sangiorgi et al., 2019]) were reported in PFAPA, but no mutation could be found consistently (Berkun et al., 2011; Dagan et al., 2010; Di Gioia et al., 2015; Kolly et al., 2013; Manthiram et al., 2020; Yildiz et al., 2021a, 2021b). Cytokine levels vary massively between flare-up and remission states and often between different studies. Several reports detected an elevation of IL-18 observed in both states of disease activity and especially high IL-6 during flare ups (Stojanov et al., 2011, 2006; Yamazaki et al., 2014). Some studies observed an elevation of IL-1β serum or mRNA levels and inflammasome-associated proteins such as caspase-1 (Brown et al., 2010; Kolly et al., 2013; Luu et al., 2020; Stojanov et al., 2006). These proinflammatory cytokines are major players in many AIDs. Increased expression of T-cell chemoattractant genes as well as high levels of cytotoxic T cells were reported in the tonsils of patients (Dytrych et al., 2015) but failed to be seen in a more recent study (Luu et al., 2020). The elevation of CXCL10, an IFN-γ‒induced T-cell chemokine, which was reported during flare ups, supports the theory of T-cell involvement (Førsvoll et al., 2013a). Wekell (2019) pointed out that the T-cell involvement in PFAPA overlaps with the time of development of an adult T helper 1 response to innate stimulations and that clinical symptoms in PFAPA often disappear around the time this T helper 1 response is established (Wekell, 2019). Recent immune pathway analysis in tonsils suggested the existence of different endotypes, but supporting functional studies are still missing (Hara et al., 2021). Because tonsillectomy provides a clinical benefit for children only, underlying pathomechanisms might differ between adults and children (Burton et al., 2019). Dissimilarities in the analysis of cytokine levels might be based on heterogeneous pathophysiology or potential involvement of currently unknown variants/mutations changing the course of the immune response.

#### Clinical signs and symptoms

PFAPA is a cause of noninfectious recurrent fever in children. It is characterized by recurrent bouts of fever lasting 1‒10 days (median = 4 days) and at least one of the cardinal symptoms of pharyngitis, cervical lymphadenitis, and/or oral aphthosis (occurring in 90, 78, and 57% of all cases, respectively) as reflected by the Marshall criteria for diagnosis (Thomas et al., 1999). Disease flares occur every 1‒12 weeks (median = 4 weeks), showing a marked periodicity without a known trigger. Other signs and symptoms patients may present with during flares are malaise, abdominal symptoms (pain, vomiting, diarrhea), arthralgia/arthritis, myalgia, headache, and skin rashes. Laboratory evaluations during flares show increased inflammatory markers, such as CRP and ESR, as well as leukocytosis with neutrophilia (Feder and Salazar, 2010; Hofer et al., 2014). PFAPA occurs with an incidence of 2.3 per 10.000 children aged up to 5 years (Førsvoll et al., 2013b). A total of 90% of all patients with PFAPA experience disease onset before the age of 5 years, with a median age of 1.7 years at onset (Hofer et al., 2014). However, adulthood onset has been reported as well (Padeh et al., 2008). In most patients, the disease resolves spontaneously before the age of 10 years (Padeh et al., 2008). About 27% of patients report a positive family history of recurrent fever, recurrent tonsillitis, PFAPA, or FMF (Hofer et al., 2014). Cyclic neutropenia, a disease in which children present with recurrent episodes of neutropenia associated with fever, malaise, lymphadenopathy, oral ulcers, and/or recurrent infections every 3 weeks, needs to be excluded to diagnose PFAPA (Lazzareschi et al., 2022).

#### Cutaneous signs

Aphthous stomatitis occurs in 57% of all patients with PFAPA during disease flares and in 6.6% even between flares (Hofer et al., 2014). Patients usually present with 1‒4 oral aphthae with a diameter of usually <1 cm. Less frequently, PFAPA presents as a cluster of very small aphthae (Feder and Salazar, 2010). In general, the aphthae are small, shallow, round, and relatively painless; are surrounded by erythema; and can be found on the nonkeratinized oral mucosa (tongue, buccal) (Figure 5b) (Femiano et al., 2008). Skin rashes were reported to occur in 13%. However, most reports lack a detailed description of these cutaneous manifestations. One report described a mildly pruritic rash consisting of erythematous papules; vesicles; and crusts on the face, trunk, and limbs (Pityriasis lichenoides et varioliformis acuta) (Iba et al., 2011).

#### Treatment

Management of PFAPA consists of two strategies: resolving flares and flare prevention. Antipyretics (e.g., acetaminophen, NSAIDs) can be given to control fever. However, remission of other disease symptoms usually requires treatment with corticosteroids (Batu, 2019). In case reports, anakinra and canakinumab have also been used successfully (Cantarini et al., 2012; Lopalco et al., 2017; Stojanov et al., 2011). For flare prevention, H2-receptor antagonists cimetidine and colchicine were found to be effective in reducing flare frequency (Butbul Aviel et al., 2016; Feder and Salazar, 2010). (Adeno)tonsillectomy is an effective treatment option for PFAPA but requires careful consideration of the risks and consequences of surgery against other noninvasive treatment regimens (Burton et al., 2019).

### SchS

Current data on cytokine levels suggest an underlying myeloid inflammation in SchS, with no association with other AID-associated mutations such as *NLRP3*, *NRLC4*, or *TNFRSF1A* (Masson Regnault et al., 2020; Louvrier et al., 2022; Pathak et al., 2019; Rowczenio et al., 2018). A few sporadic mutations in *NLRP3* have been reported (de Koning et al., 2015; Loock et al., 2010; Rowczenio et al., 2013), but Rowczenio et al. (2018) found no evidence of *NLRP3* mutations driving the pathogenesis of SchS in a larger cohort, leading to the presumption that these patients might fall into the category of late-onset acquired NLRP3-associated AID (Rowczenio et al., 2017). A molecular mechanism shared between patients with SchS remains elusive. It is speculated that SchS could behave similarly to Waldenström’s macroglobulinemia, a disease defined by monoclonal gammopathy, where a mutation in *MYD88* was reported (van Leersum et al., 2019). *MyD88* is relevant for the signaling pathway of toll-like receptor 4 and IL-1 receptor and causes the induction of the transcription factor NF-kB (Treon et al., 2012). The increase of a signaling factor such as NF-kB could explain the interplay of the dysregulated innate immune system and the hypogammaglobinemia without mutation of inflammasome sensors. Indeed, the *MyD88* L265P variant was found in 9 of 30 patients tested, making it the most common genetic variant found in SchS (Pathak et al., 2019). Similar to other polygenic AIDs, high levels of IL-6 are associated with the activity of the disease (de Koning et al., 2007; Pizzirani et al., 2009). Elevated IL-18 and IL-1β, products of inflammasome-induced caspase-1 cleavage, as well as high levels of ASC specks in serum, were reported (Migliorini et al., 2009; Pizzirani et al., 2009; Rowczenio et al., 2018). IL-β proves to be a poor indicator of disease activity because measurement of circulatory IL-1β remains difficult, which could explain reports with no IL-1β in sera of patients (Kluger et al., 2009; Migliorini et al., 2009). PBMCs of patients showed an elevation of spontaneous release of IL-1β, IL-6, and TNF-α and increased production of these factors after stimulation (Masson Regnault et al., 2020). Masson Regnault et al. (2020) reported additional cytokine studies indicating T-cell immunosuppression in SchS. Similar aspects of T-cell involvement, especially Th17 cells, were reported earlier (Noster et al., 2016), and a connection between IL-1β and T-cell differentiation was already reported in 2007 (Acosta-Rodriguez et al., 2007). These findings as well as the hypergammaglobinemia seen in SchS might merely be a byproduct of the innate immune system’s response or could be induced by yet unknown mechanisms. Treatment efforts to reduce the overproduction of proinflammatory cytokines such as IL-1 and IL-6 showed promising results (Claus and Vanderschueren, 2019; Kluger et al., 2009; Krause et al., 2017; Rowczenio et al., 2018).

#### Clinical signs and symptoms

SchS is a rare, late-onset AID without familial clustering, with only 300 cases being reported in the medical literature so far (de Koning, 2014). According to the diagnostic Strasbourg criteria established in 2012, SchS shows a chronic urticarial rash and monoclonal gammopathy of mostly IgM and rarely IgG (85 and 15% of cases, respectively; obligatory major criteria) (de Koning, 2014). The minor Strasbourg criteria of the disease include recurrent fever, abnormal bone remodeling with or without bone pain, a neutrophilic infiltrate on skin biopsy, and leukocytosis or elevated CRP. The diagnosis SchS is made in the presence of both obligatory criteria and additionally two (in cases of IgM gammopathy) or three (in cases of IgG gammopathy) minor criteria. In general, the urticarial skin rash is the presenting sign of disease and precedes other signs and symptoms (median age at rash onset = 51 years, median age at fever onset = 52 years) (de Koning, 2014). The frequency of cutaneous findings varies greatly, with some patients experiencing continuous skin symptoms, whereas others experience flare-up only a few times a year. Individual skin lesions persist for 12‒48 hours (de Koning et al., 2014, 2007). Flares of rashes and fever commonly occur at the same time and can be triggered by stress, cold exposure, heat exposure, alcohol, spicy food, and physical activity (de Koning et al., 2007; Lipsker, 2010). In addition to the Strasbourg criteria, patients may experience arthralgia, myalgia, lymphadenopathy, weight loss, hepatosplenomegaly, and angioedema (de Koning, 2014). The risk of developing a lymphoproliferative disorder, mostly Waldenström’s macroglobulinemia, within 10 years is approximately 13% (de Koning et al., 2014, 2007). Laboratory findings include an increase in CRP and ESR, leukocytosis with neutrophilia, and anemia (de Koning, 2014).

#### Cutaneous signs

**Figure 6:**
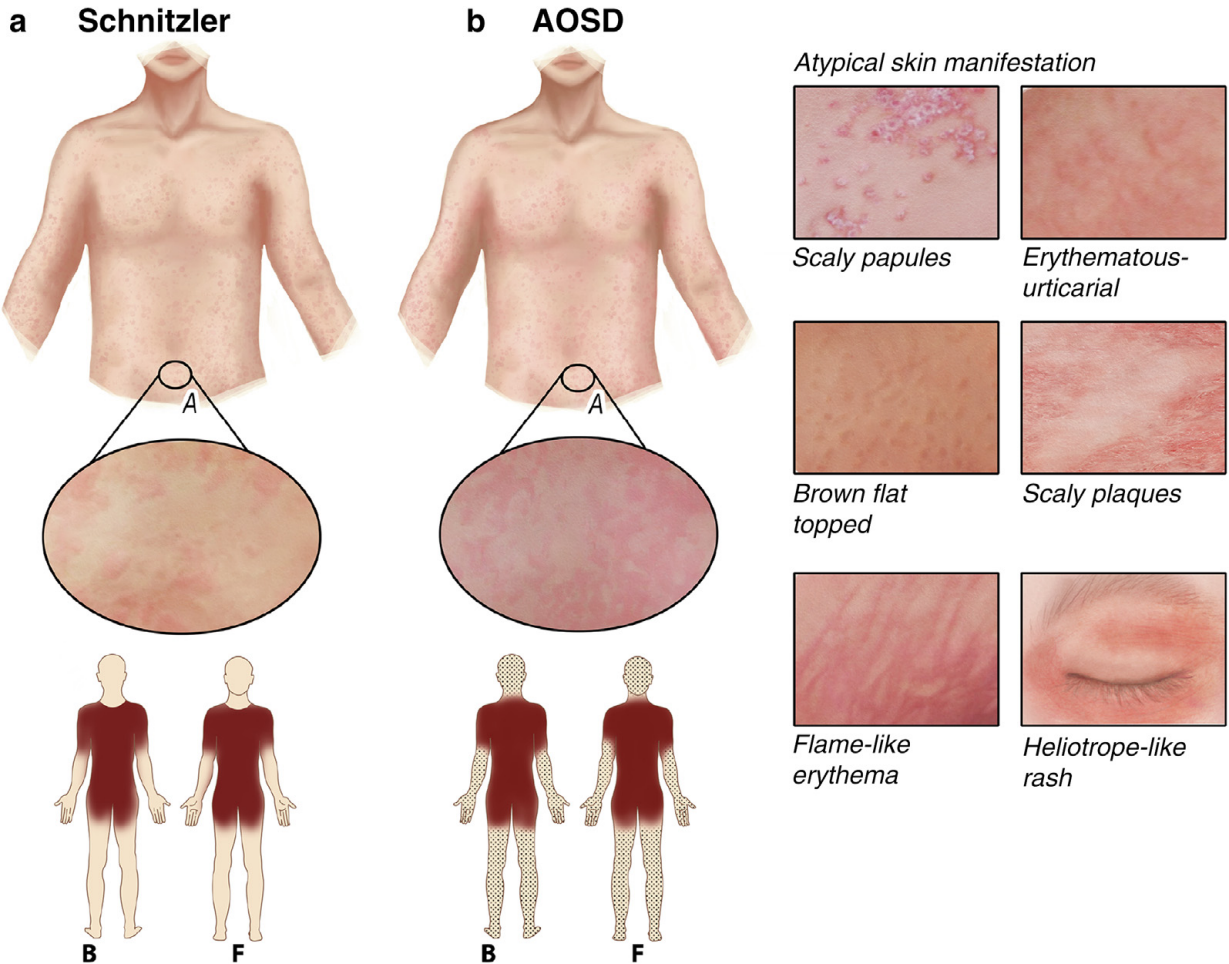
**Cutaneous signs of Schnitzler and AOSD.** (**a**) SchS: chronic urticaria with sometimes confluent, erythematous lesions in symmetrical distribution on trunk and extremities. (**b**) AOSD: typical salmon-colored macular or maculopapular rash on the trunk and/or proximal limb (left). Atypical rashes (right): scaly papules partly coalescing into erythematous plaques, erythematous-urticarial lesions, brown-flat-lichenoid lesions, flame-like erythema, and heliotrope-like rash of the eyelids. Other manifestations (e.g., acne, angioedema) are not shown. B denotes the back view, and F denotes the front view. AOSD, adult-onset Still disease; SchS, Schnitzler syndrome.

Neutrophilic urticarial dermatosis (NUD) is the hallmark cutaneous finding of SchS and often precedes other signs and symptoms of the disease (de Koning, 2014). However, NUD is not specific to SchS because it can also occur in other autoinflammatory syndromes such as CAPS and AOSD, which need to be ruled out as differential diagnoses (Chen et al., 2022; Gusdorf and Lipsker, 2020). NUD is characterized by sometimes confluent, erythematous, annular, or maculopapular lesions with a diameter between 0.5 and 3 cm. Compared with chronic spontaneous urticaria, SchS lesions are less edematous. The rash is symmetrically distributed and involves the trunk and extremities with only rare involvement of the head and neck. The palms and soles are never affected (Figure 6a). The frequency of skin flares varies considerably from daily to only a few times a year, whereas single-skin lesions usually last 12‒48 hours and heal without scaring (de Koning et al., 2014, 2007). Cutaneous involvement was reported to be triggered by various factors such as stress, alcohol, spicy, food, physical work, and exposure to hot or cold temperatures (de Koning et al., 2007; Lipsker, 2010). However, the ice cube test is negative (Krause et al., 2012b). Only 21% of patients with SchS develop pruritic skin lesions over time, with patients commonly reporting a burning sensation instead. Angioedema occurs in 8% of patients with SchS only (de Koning, 2014).

#### Treatment

Antihistaminic drugs, colchicine, NSAIDs, and corticosteroids are usually not or only partly beneficial. Anti‒IL-1 treatment with anakinra, rilonacept, or canakinumab has been shown to be very effective (Krause et al., 2017; Néel et al., 2014). For patients who do not respond to anti‒IL-1 treatment, the IL-6 inhibitor tocilizumab can be considered (Bonnekoh et al., 2021; Claus and Vanderschueren, 2019; Krause et al., 2012a).

### AOSD

AOSD presents with an elevation of various cytokines, which underlines the autoinflammatory component of the disease. Several trigger factors are discussed, and multiple genetic alleles raising the susceptibility to the disease have been reported. HLA molecules are known to be associated with many rheumatologic diseases, and although large cohort studies in AOSD are rare, associations with different *HLA* alleles and other genetic variants have been reported (Asano et al., 2017; Joung et al., 2003). Recently Teng et al. (2021) connected variants in HLA II molecules (most prominent *HLA-DRB1* and *HLA-DQA1/B1*) of 264 patients to AOSD in the Han Chinese population. In addition, variants in *MEFV* and *TNFRSF1A* could be observed in a cohort of 40 patients with AOSD in Germany (Sighart et al., 2018). A novel SNV in the gene coding for the macrophage colony-stimulating factor (M-CSF), a known hematopoietic GF involved in the differentiation and stimulation of monocytes/macrophages, was associated with high levels of M-CSF in the plasma of patients with AOSD (Chen et al., 2020; Nemunaitis, 1993). Another polymorphism targeting neutrophil activation was recently linked to the functional *LILRA3* to leukocytosis and neutrophilia in AOSD (Wang et al., 2021). *LILRA3* mRNA expression can be linked to disease activity and circulating neutrophil extracellular traps (NET)‒DNA complexes (Wang et al., 2021). The real impact of these mutations on the wide heterogeneity of AOSD seen in clinical practice still needs to be explored. Next to the elevation of M-CSF production (Matsui et al., 1999) and NET formation (Hu et al., 2019), an increase of several other chemokines and cytokines was observed. IL-1, IL-6, IL-8, IL-18, TNF-α, as well as sTNFR2 are the most prominent among them (Choi et al., 2003; Feist et al., 2018; Tang et al., 2021). Markedly elevated cytokines and hyperferritinemia are associated with macrophage activation syndrome (MAS), a life-threatening reaction of the innate immune system resulting in a cytokine storm (Inoue et al., 2016; Wang et al., 2020). MAS is a major cause of death in patients with AOSD, complicating around 15% of the cases, with an overall ratio of 7:3 (female:male) (Wang et al., 2020). Attempts to cluster the wide heterogeneity of clinical presentations seen in AOSD highlight a subgroup with a MAS occurrence rate of 54% mostly consisting of patients with older age and overall high systemic scores (>7) (Berardicurti et al., 2021). The active phase of the disease shows very high levels of IL-18, a proinflammatory cytokine that influences cell adhesion and chemokine production and is important for the regulation of NK cell activity (Kaplanski, 2018). IL-1β and IL-18 are released in great amounts after inflammasome activation, and elevated NLRP3-inflammasome expression could be positively correlated to the disease activity (Hsieh et al., 2017). Interestingly, several bacterial, viral, and endogenous triggers have been reported in AOSD (Jung et al., 2020). Hu et al. (2019) connected the enhanced formation of NETs in AOSD to increased activation of the NLRP3 inflammasome. Neutrophils, monocytes, and macrophages were described as the driving factors behind the pathogenesis of the disease, but cytokine studies connecting the clinical heterogeneity to different pathogenic processes are still missing. These studies would be needed to validate potential biomarkers and help to establish a promising treatment plan.

#### Clinical signs and symptoms

The four cardinal symptoms of AOSD are quotidian or double-quotidian fever up to 39 °C for at least 1 week, a concomitant skin rash, leukocytosis with neutrophilia, and arthralgia/arthritis lasting for at least 2 weeks, as reflected by the Yamaguchi criteria and the modified criteria published by Fautrel et al. in 2002 (Fautrel et al., 2002; Yamaguchi et al., 1992). During fever flares, which persist for some hours, patients experience malaise. Other symptoms include lymphadenopathy, a sore throat, splenomegaly, myalgia, pleural effusion, and/or pericarditis (Asanuma et al., 2015). AOSD can also involve life-threatening complications such as MAS, disseminated intravascular coagulopathy, fulminant hepatitis, cardiac involvement, acute respiratory distress syndrome, and pulmonary arterial hypertension (Efthimiou et al., 2014; Feist et al., 2018). As the name implies, AOSD has its disease onset in adulthood, with a median age of 46 years at diagnosis and a reported prevalence between 1 and 39 per one million (Asanuma et al., 2015; Wakai et al., 1997). Bacterial and viral infections have been hypothesized to contribute to the onset of disease, with viral infections commonly suggested as a trigger for relapse (Feist et al., 2018; Jia et al., 2019). However, no risk factors or triggers have been identified for single flares so far. Besides leukocytosis with neutrophilia, common laboratory findings include increased ESR, elevated levels of CRP, elevated ferritin (indicating macrophage activation), elevated transaminases, as well as decreased serum albumin (Asanuma et al., 2015).

#### Cutaneous signs

AOSD is associated with cutaneous involvement in 80% of patients, which can be divided into two subgroups: a typical and an atypical rash (Figure 6b). A total of 86% of patients present with the typical evanescent and salmon-colored macular or maculopapular rash on the trunk and/or proximal limbs, rarely involving the head, palms, or soles. It usually develops in the late afternoon during fever spikes and vanishes without scar formation and is commonly associated with a mild itch or burning sensation (Cozzi et al., 2016). In contrast, 78% of the patients present with the atypical rash. Both typical and atypical skin manifestations can occur concomitantly (Lee et al., 2012). The atypical skin rash encompasses a wide range of different lesions. The most common atypical skin manifestation is characterized by widespread, pruritic, scaly, and persistent papules, partly coalescing into erythematous plaques on the head, trunk, and extensor sides of the limbs. The skin lesions can also be erythematous urticarial; brown-flat lichenoid in linear distribution, possibly representing the Koebner phenomenon, and dermatomyositis like with a heliotrope-like rash of the eyelids. Other reported skin findings among the group of atypical skin manifestations include acne-like rashes, vesiculopustular lesions, fixed plaques, angioedema, generalized erythema, and flagellate erythema (Cozzi et al., 2016; Lee et al., 2012; Yamamoto, 2012).

#### Treatment

Treatment choices include NSAIDs, glucocorticoids, and targeted therapy. NSAID monotherapy leads to disease control in only 7‒15% of patients; thus, patients usually require additional treatment with corticosteroids (Efthimiou et al., 2006; Franchini et al., 2010). Targeted therapy is required in a third of patients whose disease is only controlled inadequately under NSAID and/or glucocorticoid treatment, with IL-1 inhibitors being the treatments of choice (anakinra, canakinumab). The IL-6 antagonist tocilizumab can be used as an alternative treatment approach, whereas TNF-α inhibitors are considered only the third line showing less promising results in trials (Feist et al., 2018).

## Concluding Remarks

The skin is the outmost barrier of the human body. Similar to a painting on a canvas, disease processes can be visible on the skin, and pattern recognition of signs and symptoms helps physicians to steer further workup, diagnosis, and treatment. The group of AIDs is a constantly growing field emphasizing the importance of the innate immune system for the body. Disruption of the TNF pathways leads to TRAPS (migratory, erythematous patches and plaques), whereas various changes in the IFN pathways lead to AIDs, including CANDLE (lipodystrophy, violaceous plaques with raised borders, violaceous swellings of the lips and eyelids) or SAVI (violaceous, ulcerating plaques; facial erythema; telangiectatic lesions; tissue loss). The large IL-1 family is involved through their regulatory mechanism of receptor antagonists whose disruption leads to DITRA or DIRA (erythema studded with pustules). Because patients with AID might seek medical advice from physicians of different specialties, the knowledge of AID-associated skin signs is an indispensable puzzle piece in the diagnosis of these rare diseases.

### Ethical approval information

Ethical approval was not applicable. Patients have given consent for the publication of their photographs.

### Data availability statement

No datasets were generated or analyzed during this study.

## ORCIDs

Dörte Symmank: http://orcid.org/0000-0002-3992-6447

Carina Borst: http://orcid.org/0000-0002-3451-7820

Mathias Drach: http://orcid.org/0000-0002-0448-1978

Wolfgang Weninger: http://orcid.org/0000-0003-3133-8699

## Author Contributions

Conceptualization: DS, CB, WW; Data Curation: DS, CB; Supervision: WW; Visualization: DS; Writing - Original Draft Preparation: DS, CB, MD, WW; Writing - Review and Editing: DS, CB, WW
